# Family resilience in a social-ecological context – emotional difficulties and coping strategies

**DOI:** 10.3389/fpsyg.2024.1421745

**Published:** 2024-07-01

**Authors:** Elena Otilia Vladislav, Gabriela Marc, Corina-Ioana Paica, Ovidiu Pop

**Affiliations:** Department of Applied Psychology & Psychotherapy, Faculty of Psychology and Educational Sciences, University of Bucharest, Bucharest, Romania

**Keywords:** family resilience, well-being, parents, coping, emotional difficulties, adaptability

## Abstract

**Introduction:**

This research explored how crises such as the pandemic influence the family dynamic and the way that the parents and the children face new difficulties and challenges. The present study investigates children’s and young people’s emotional states, the dimensions of family resilience, and the types of coping strategies and parents’ emotional states during the coronavirus pandemic. The final sample for the research was represented by 1,010 parents from Romania.

**Methods:**

The present scientific research is a transversal study with the scope to understand the emotional difficulties that parents and children/ young people face and the coping strategies that they adopt in a crisis, such as the time of the coronavirus pandemic.

**Results:**

Parents with maladaptive coping strategies are more affected by the pandemic. Also, the results indicate that there is a significant correlation between parental coping strategies and the children’s emotional states, and the adaptability level. Therefore, the high scores on the subscales which measure maladaptive coping indicate high scores of the levels of depression, anxiety, and stress of the parents. Family resilience, through the dimension of ‘communication and problem-solving in the family’ has a moderation role in the relationship between parental depression and the maladaptive coping strategy of rumination. Family resilience through ‘maintaining a positive attitude’ has a moderation role in the relationship between catastrophizing and the teenagers’ level of adaptability.

**Discussion:**

The results of the study pointed out the role and place of family resilience within the family system and how a period of crisis can affect this system.

## 1 Introduction

In developmental psychology and mental health theory, research, and practice, the idea of resilience has gained prominence, challenging the predominant focus on dysfunction and disorder. The ability to endure and overcome significant adversity in life is known as resilience (Piotrowski et al., 2021). According to Luthar (2006), resilience is the result of dynamic processes that promote adaptive growth in the face of severe adversity. These resources and strengths facilitate healing and development in addition to coping and adjustment. Prime et al. (2020) note that the precise impact of the pandemic on family well-being remains uncertain in their paper on risks and family resilience during the pandemic. However, over a third of families reported experiencing severe anxiety from the stress of isolation during the pandemic (Statistics Canada, 2020).

Over time, the family occupied a special place in research and several explanatory models appeared. Don Jackson (Ray, 2009) defined the family as a system with its own homeostasis that allows it to resist changes, while Bowen and Jahangiri (2019) considered the family from a psychodynamic perspective. He affirms that the family is a system of emotional relationships. Often, in his approach, he focused on one family member and how he or she relates to the rest of the family. Minuchin (1998), representative of structural family therapy, considered that the family is more than a sum of individuals and that within it a series of interactions take place according to some rules, explicit or not, and the totality of these rules constitutes a structure. Family is relevance to individual and social functioning. The family ensures human development, stability, the fulfillment of objectives and socio-emotional support, strengthens mental health and generates resources for the formation of individual and family resilience.

The way a family adapts to new challenges and copes with adversities, such as stress, crisis, and threats during uncertain times, such as the COVID-19 pandemic, is referred to as resilience. Family resilience is also defined as the ability to withstand and recover from adversity, which necessitates constructive adaptation, with resistance to losses and the ability to face unexpected difficulties that arise and are sometimes beyond the individual’s control (Walsh, 2020).

Walsh (2015) elucidates the intricate interplay between biopsychosocial factors, risk, and resilience, highlighting key transactional processes that help struggling families become more resilient and stronger. These key processes in family resilience are belief systems (making meaning of adversity, positive outlook, transcendence, and spirituality), organizational processes (flexibility, connectedness, mobilize social and economic resources), communication and problem-solving processes (clarity, open emotional sharing, collaborative problem solving) (Walsh, 2016a,b).

There are various benefits to using a family resilience framework (Walsh, 2016a,b). It first, by definition, concentrates on strengths developed in times of stress, in response to crisis, and during prolonged adversity. Second, it is considered that no family or set of circumstances can fit neatly into a single model of healthy functioning. Functioning is evaluated in the context of each family’s values, structural, situational, and relational resources, and constraints, as well as the challenges it faces. Third, when problems develop and families grow over their life cycle and across generations, procedures for optimal functioning and member well-being change with time.

If until now the studies focused either on adults (Marzilli et al., 2021), or on young people (Guessoum et al., 2020) and children (López-Bueno et al., 2021), this study follows the family, through the way parents use coping strategies and their impact on the emotional health of children. As concern the child’s self-regulation this is determined by the more remote factors’ influence, for example, social disturbances due to the pandemic and proximal processes, the relationships with those close to them, such as family members, teachers, and peers (Haine-Schlagel and Walsh, 2015; Browne et al., 2016). Therefore, understanding how one family member’s functioning impacts another family member’s functioning is essential for understanding the effect of a crisis on the family’s well-being.

Li and Li (2021) described family resilience as the collective capability of family members to navigate and overcome challenging situations, stressors, and adversities. This involves a family’s ability to rebound from life transitions and crises through warmth, support, and cohesion. Such positive behaviors and strategies exhibited by family members enable them to quickly recover from crises, ensuring the ongoing functioning and development of the family unit (Zhao et al., 2023). Family resilience is a multifaceted process that includes interactions between families and other systems within complex environments, which enhances the family’s ability to cope with adversity over time (Ungar, 2015).

During the COVID-19 pandemic, both individual and family resilience have been crucial in supporting positive coping mechanisms in the face of adversity (Chan et al., 2021). By understanding and fostering family resilience, families can adapt, thrive, and maintain competent functioning following significant adversities or crises (Patterson, 2002).

Ying et al. (2020) indicated that the COVID-19 pandemic has led to mental and health problems, including stress, anxiety, and depression. Higher family resilience was associated with lower levels of anxiety, stress, and depression. The outbreak has particularly impacted the mental health of family members of healthcare workers, causing stress and anxiety (Ying et al., 2020).

The COVID-19 pandemic has brought both positive and negative effects on families. While it has led (in some cases) to increased quality time spent together, it has also caused disruptions in family relationships (Luttik et al., 2020). Parents play a crucial role in fostering family resilience, especially during large-scale public health crises. The resilience of children and adolescents is significantly influenced by their parents’ resilience, including the level of care parents provide for themselves and for their families (Luthar et al., 2021). To prevent significant distress among young individuals facing high levels of stress, interventions should not only focus on the mental health of children, but also, on supporting key caregiving adults at home and in educational settings. Positive adaptation by parents during the pandemic can positively impact children’s adaptation.

Another authors (Black and Lobo, 2008) consider that a repertoire of possible coping factors is exchanged in resilient families. There are times when family demands exceed the family’s capabilities. When these imbalances exist, some capabilities may supersede others toward regaining equilibrium.

Like the previous studies, we also consider in this study the fact that an important factor in strengthening the family’s resilience during a significant health crisis is the parents. The resilience of children and adolescents is impacted by the way parents care for their families and themselves. Children may develop positive adaptation skills because of their parents’ positive pandemic adaption. Access to parental support is critical for mitigating the negative impact of COVID-19 on family resilience (Salin et al., 2020; Gayatri and Irawaty, 2022).

Familial coping is a cognitive strategy used by families to deal with stressful situations. Recent research (Killgore et al., 2020; Salin et al., 2020) found that families have developed more ways to cope during the COVID-19 pandemic and quarantine: increased support perceived by the family, increased social support perrelationship, the from the friends, less severe insomnia episodes, increased care and support from a close relationship, the faith, the hope and optimism, flexibility, financial management, communication, leisure time spent in the family. According to the findings of Mashudi and Yusuf (2021) the adaptation strategy utilized by the family during the COVID-19 pandemic accounts for 15% of the family’s health. It is anticipated that the family will assist in emotional problem-solving by providing useful coping mechanisms. In this study we follow what kind of strategies parents use in crisis situations and how these strategies emotionally influence parents and their children.

The primary goal of this study is to investigate how crises such as the pandemic affect the family dynamic and how parents and children deal with new difficulties and challenges.

At the same time, the present research has more specific objectives, such as:

1. The identification of the emotional states of parents and the types of coping mechanisms they employed throughout the coronavirus pandemic.
2. Knowing the emotional status of children and teenagers during the coronavirus pandemic as assessed by their parents, including anxiety, depression, somatization, and adaptation.
3. Identifying the relationship between the maladaptive coping strategies used by the parents and how they affect children’s emotional health and level of adaptability.
4. Establishing the moderating role of family resilience in the relationship between parents’ emotional state and coping strategies, and the level of adaptation of adolescents.

According to these objectives, the following hypotheses were put forward:

1. Maladaptive coping strategies generate high levels of depression, anxiety, and stress of the parents.
2. The emotional and adaptation difficulties of the children are associated with the maladaptive coping strategies of the parents.
3. Family resilience has a moderating role in the relationship between parents’ depression and rumination, as a maladaptive coping strategy.
4. Family resilience has a moderating role in the relationship between the adaptation level of adolescents and catastrophizing, as a maladaptive coping strategy.

## 2 Materials and methods

### 2.1 Sample

The final sample for the research was represented by 1,010 adults (82% female and 18% male) with an average age of 40 and standard deviation of 6.02. All participants are parents, and their children are in the age categories: 2–6 years (26%), 6–12 years (43%), and 12–18 years (31%). The sample is predominantly composed of individuals hailing from urban areas, constituting 88.7% of the total. Within this group, 60% are actively employed and 40% have their own business. In terms of marital status, a significant 79.1% are married, 10.4% have experienced divorce and 10.5% of them have a children and a partner. The children were grouped into three categories according to the application standards of the evaluation tool Behavior Assessment System for Children Second Edition (Reynolds and Kamphaus, 2004). This study names children those aged between 2 and 6 years, pre-teenagers those aged between 6 and 12 years and teenagers those aged between 12 and 18 years. In the analyzes carried out, we looked for associations between parents’ coping strategies and children’s, pre-teenagers, and teenagers’ emotional difficulties.

The selection method was sampling by convenience, which implies that the sample includes subjects that are accessible and available. The usefulness of this method cannot be denied when the special and temporal context of the selection is not directly linked with the dependent variable. If the subject’s availability is not affected by any aspect which could significantly influence the scope of the research, then the sample is accepted.

### 2.2 Measures

The present scientific research is a transversal study with the scope to understand the emotional difficulties that the parents and the children/ young people face and the adaptive strategies that they adopt in a crisis, such as the time of the coronavirus pandemic. The participants completed self-evaluation questionnaires and an evaluation questionnaire of their children.

#### 2.2.1 Emotional difficulties

Depression Anxiety Stress Scales (Lovibond and Lovibond, 1995)- DASS is an instrument used clinically and in research (Osman et al., 2012) for the assessment and structuring of three areas of suffering: symptoms of depression, symptoms of anxiety, and general stress. We used in our study the 21-item form (DASS-21R) which was adapted and standardized for the Romanian population by Perțe (2011). The DASS contains items such as: “It seemed to me that I was not able to mobilize myself at all,” “I was not able to get excited about anything.”

The response method is on a Likert scale with values from 0 to 3, with the following response options: 0 - It did not suit me, 1 - It suited me to some extent or from time to time, 2 - It suited me quite a lot or quite often, 3 - It suited me a lot or almost all the time.

For the present study the coefficients for the internal consistency for each subscale were: *α* = 0.80 for depression, *α* = 0.68 for anxiety and *α* = 0.82 for stress.

#### 2.2.2 Coping strategies

Cognitive Emotional Response Questionnaire (Garnefski and Kraaij, 2007) – CERQ consists of 36 items for assessing nine strategies for cognitive emotion regulation. CERQ is made up of two factors. The first factor, called cognitive emotion regulation orientated toward the positive, is in theory more adaptive, consisting of subscales: positive reassessment, putting in perspective, positive refocusing, planification, and acceptance. The second factor refers also to cognitive emotion regulation but is focused on the negative, and consists of these sub-scales: self-blame, other blame, rumination, and catastrophizing. Cognitive Emotional Response Questionnaire was adapted and standardized for the Romanian population by Perțe and Miclea (2010). The CERQ contains items such as: “I think that I have to accept what happened,” “I think about pleasant things that have nothing to do with the situation,” “I think that what happened to me is the worst thing that can happen to anyone.”

The answer method is on a Likert scale with values from 1 to 5, with the following answer options: 1- (almost) never, 2- sometimes, 3- usually, 4- often, 5- (almost) every time.

Regarding the values for the coefficient Cronbach Alpha for our study, these are self-blame *α* = 0.81, acceptance *α* = 0.92, rumination *α* = 0.76, positive refocusing *α* = 0.70, putting in perspective *α* = 0.91, positive reassessment *α* = 1, planification *α* = 1, catastrophizing *α* = 1, other-blame *α* = 0.89.

#### 2.2.3 Children behavior

Behavior Assessment System for Children Second Edition (Reynolds and Kamphaus, 2004) – BASC-2 is a well-known system, used by psychologists, specialists from the educational system, doctors, and other clinicians, to find out more information about the child’s behavior and feelings. Being developed by remarkable experts in child behavior, BASC-2 contains more components that gather information from the parents, teachers, and the child. This information focuses on the areas of strengths and weaknesses in the child’s behavior and feelings, in a way that the child’s strengths do not go unnoticed, while the potentially problematic domains are investigated.

During the research, we used the assessment method BASC-2 because this is the latest standardized version used on the Romanian population. Behavior Assessment System for Children Second Edition was adapted and standardized for the Romanian population by Mitrofan et al. (2011). The BASC-2 was applied to parents, it contains scales answered by parents of preschoolers aged 2–5 (PRS-P), parents of children aged 6–11 (PRS-C) and parents of adolescents aged 12–18 years (PRS-A). For the present study we investigated only the perception of the parents about the observable behavior of children. BASC contains items for ages 2–5 years, such as: “Sometimes sick,” “Worries about what parents think”; for ages 6–11 years: “Cries easily,” “He/she is afraid,” “He/she adapts well to changes in his/her routine activities”; for ages 12–18: “He/she adapts well to changes in plan,” “He/she is negative,” “He/she changes his emotional state quickly.” The answer options are: never (1), sometimes (2), often (3), always (4).

The Cronbach Alpha values for the subscales from our current study are Adaptability PRS-P *α* = 0.739, Anxiety PRS-P *α* = 0.844, Depression PRS-P *α* = 0.813, Somatization PRS-P *α* = 0.816, Adaptability PRS-C *α* = 0.590, Anxiety PRS-C *α* = 0.879, Depression PRS-C *α* = 0.825, Somatization PRS-C *α* = 0.822, Adaptability PRS-A *α* = 0.733, Anxiety PRS-A *α* = 0.879, Depression PRS-A *α* = 0.849, Somatization PRS-A *α* = 0.874.

#### 2.2.4 Family resilience

Family Resilience Assessment Scale (Tucker Sixbey, 2006) – FRAS is a psychological assessment instrument that measures the level of family resilience based on the theory of Walsh (1996, 2003, 2007). The original scale’s total score ranges from 54 to 270.

The six factors in the shortened version of the FRAS correspond to the six subscales: Communication and problem-solving in the family, Social, and economic resources, Maintaining a positive attitude, Familial closeness, Family spirituality, and Ability to find meaning in difficulties. The six factors are distributed as follows: the first factor has 27 items, the second factor has 8 items, the third factor has 6 items, the fourth factor has 6 items, 4 of which are items with inverted scores, the fifth factor has 4 items, and the sixth factor has 3 items. Family Resilience Assessment Scale was adapted and standardized for the Romanian population by Bostan (2014). The FRAS contains items scored on a Likert scale from 1 to 5, where 1 – never true, 2 – somewhat true, 3 – sometimes true, 4 – mostly true, 5 – always true. Examples of items are: “We accept stressful times as part of our life,” “We are open to new ways of doing things in our family.”

Cronbach Alpha coefficients for the current study have been: 0.82 for Communication and problem-solving in the family, 0.65 for Utilizing social and economic resources, 0.57 for Maintaining a positive attitude, 0.54 for Familial closeness, 0.63 for Family spirituality and 0.61 for Ability to find meaning in difficulties.

For data analysis in moderation we used these scales in the research: Communication and problem-solving in the family and Maintaining a positive attitude. The total score for those scales vary between 33 and 132.

### 2.3 Study variables

The research variables are represented by the psychological concepts measured through the test’s subscales. As variables, there are:

- Depression, anxiety, and stress are measured with the DASS instrument,
- Self-blame, acceptance, rumination, positive refocusing, planification, positive reappraisal, putting in perspective, catastrophizing, other blame measured by CERQ instrument (however, in the current study, our focus was on the parents’ maladaptive coping strategies),
- Communication and problem-solving in the family and Maintaining a positive attitude are measured with the evaluation scale for family resilience, FRAS and
- The adaptability, anxiety, depression, and somatization subscales from the BASC-2 instrument.

### 2.4 Procedure

Participants have been recruited and measurements have been completed between April 2020 and February 2021.

Among the conditions for the study, there has been voluntary participation in all parts of the research, accessing all the tests through the Google Forms platform. There have not been exclusions based on psychological and medical status.

Before starting the research, the aim of the study has been explained to all participants, the fact that their involvement is entirely voluntary, storage, confidentiality, and keeping of data. To participate in the study, all subjects provided informed consent.

### 2.5 Analytical approach

This study corresponds to the generic model of non-experimental research, following the methods of a quantitative and cross-sectional analysis, using questionnaires, and generating quantitative data. Correlation analyzes and moderation analyzes will be pursued.

The data is collected through the Google Forms platform, and the order of presentation of the tools is Behavior Assessment System for Children Second Edition, Cognitive Emotional Response Questionnaire, Depression Anxiety Stress Scales, Family Resilience Assessment Scale. There were no special situations in the data collection procedure.

The data analysis will be carried out using the SPSS program. There were no special situations (e.g., outliers) in data collection.

## 3 Results

The results include the fulfillment of the objectives and refer to Parental emotional difficulties, Children’s emotions and parental coping strategies and Family resilience.

### 3.1 Parental emotional difficulties

Table 1 presents the mean, the range and the standard deviation of the psychological dimensions measured through testing the parents, specifically the parents’ emotional states and the types of coping strategies when faced with a crisis. Table 2 presents the Pearson correlation for depression, stress, anxiety, and coping strategies.

**Table 1:** Descriptive statistics.

	*N*	*M*	SD
Depression		4.61	4.33
Anxiety		4.43	4.86
Stress		7.81	5.48
Self-blame		11.29	3.58
Acceptance		14.08	3.30
Rumination		14.55	3.71
Positive refocusing		13.69	3.84
Planification		16.83	2.85
Positive reassessment		16.72	3.10
Perspective		15.63	3.49
Catastrophizing		9.13	3.49
Blaming others		8.30	3.19
Total	1,010		

**Table 2:** Parental emotional difficulties and maladaptive coping strategies.

		Depression	Anxiety	Stress	Self-blame
Depression	Pearson Correlation	1	0.761^**^	0.753^**^	0.283^**^
	Sig. (two-tailed)		0.000	0.000	0.000
	*N*	1,010	1,010	1,010	1,010
Anxiety	Pearson Correlation	0.761^**^	1	0.754^**^	0.252^**^
	Sig. (two-tailed)	0.000		0.000	0.000
	*N*	1,010	1,010	1,010	1,010
Stress	Pearson Correlation	0.753^**^	0.754^**^	1	0.322^**^
	Sig. (two-tailed)	0.000	0.000		0.000
	*N*	1,010	1,010	1,010	1,010
Blaming other	Pearson Correlation	0.292^**^	0.282^**^	0.328^**^	0.179^**^
	Sig. (two-tailed)	0.000	0.000	0.000	0.000
	*N*	1,010	1,010	1,010	1,010
Rumination	Pearson Correlation	0.137^**^	0.161^**^	0.203^**^	0.435^**^
	Sig. (two-tailed)	0.000	0.000	0.000	0.000
	*N*	1,010	1,010	1,010	1,010
Catastrophizing	Pearson correlation	0.409^**^	0.402^**^	0.395^**^	0.342^**^
	Sig. (two-tailed)	0.000	0.000	0.000	0.000
	*N*	1,010	1,010	1,010	1,010

**p < 0.01.

Parents with maladaptive coping strategies are more affected by the pandemic, therefore the high scores on the subscales which measure maladaptive coping indicate high scores of the levels of depression, anxiety, and stress. High scores on the depression scales are positively linked to all the maladaptive coping strategies (self-blame *r* = 0.283, *p* = 0.000; blaming other *r* = 0.292, *p* = 0.000; rumination *r* = 0.137, *p* = 0.000; catastrophizing *r* = 0.409, *p* = 0.000). The coefficient of determination r^2^ values range between 0.018 and 0.167.

High scores for anxiety are linked to maladaptive coping strategies (self-blame *r* = 0.252, *p* = 0.000; blaming other *r* = 0.282, *p* = 0.000; rumination *r* = 0.161, *p* = 0.000; catastrophizing *r* = 0.402, *p* = 0.000). The coefficient of determination *r*^2^ values range from 0.025 and 0.161.

High level of stress is linked to high scores on all the subscales for maladaptive coping strategies (self-blame *r* = 0.322 *p* = 0.000; blaming others *r* = 0.328, *p* = 0.000; rumination *r* = 0.203, *p* = 0.000; catastrophizing *r* = 0.395, *p* = 0.000). *r*^2^ values vary between 0.041 and 0.156.

In conclusion, individuals with maladaptive coping mechanisms had higher levels of stress, anxiety, and depression (Table 2).

### 3.2 Children’s emotions and parental coping strategies

The research’s findings show positive significant correlations, between the children’s emotional states and the maladaptive coping mechanisms used by their parents, and a negative link between teenagers’ degree of adaptability and the maladaptive coping strategies. The significant correlations observed, in the case of each of the three age groups investigated: 2–6 years (children), 6–12 years (pre-teenagers), and 12–18 years (teenagers) are presented in Tables 3–6.

**Table 3:** Children, pre-teenagers and teenagers’ depression and maladaptive coping strategies used by parents.

		depression_children	depression_preteenagers	depression_ teenagers	self-blame
depression_children	Pearson Correlation	1	−0.320^**^	−0.233^**^	0.128^**^
	Sig. (two-tailed)		0.000	0.000	0.000
	*N*	1,010	1,010	1,010	1,010
depression_preteenagers	Pearson Correlation	−0.320^**^	1	−0.314^**^	0.120^**^
	Sig. (two-tailed)	0.000		0.000	0.000
	*N*	1,010	1,010	1,010	1,010
depression_teenagers	Pearson Correlation	−0.233^**^	−0.314^**^	1	0.068^*^
	Sig. (two-tailed)	0.000	0.000		0.030
	*N*	1,010	1,010	1,010	1,010
Blaming other	Pearson Correlation	0.075^*^	0.059	0.025	0.179^**^
	Sig. (two-tailed)	0.017	0.061	0.420	0.000
	*N*	1,010	1,010	1,010	1,010
Rumination	Pearson Correlation	0.121^**^	0.003	−0.007	0.435^**^
	Sig. (two-tailed)	0.000	0.930	0.830	0.000
	*N*	1,010	1,010	1,010	1,010
Catastrophizing	Pearson Correlation	0.063^*^	0.087^**^	0.069^*^	0.342^**^
	Sig. (two-tailed)	0.044	0.006	0.028	0.000
	*N*	1,010	1,010	1,010	1,010

**p < 0.01, *p < 0.05.

**Table 4:** Children, pre-teenagers and teenagers’ anxiety and maladaptive coping strategies used by parents.

		anxiety_children	anxiety_preteenagers	anxiety_teenagers	Self-blame
anxiety_children	Pearson correlation	1	−0.334^**^	−0.253^**^	0.187^**^
	Sig. (two-tailed)		0.000	0.000	0.000
	*N*	674	560	560	674
anxiety_preteenagers	Pearson correlation	−0.334^**^	1	−0.391^**^	0.160^**^
	Sig. (two-tailed)	0.000		0.000	0.000
	*N*	560	736	560	736
anxiety_teenagers	Pearson correlation	−0.253^**^	−0.391^**^	1	0.061
	Sig. (two-tailed)	0.000	0.000		0.110
	*N*	560	560	697	697
Rumination	Pearson correlation	0.164^**^	0.052	−0.001	0.435^**^
	Sig. (two-tailed)	0.000	0.158	0.987	0.000
	*N*	674	736	697	1,010
blaming other	Pearson correlation	0.079^*^	0.062	0.010	0.179^**^
	Sig. (two-tailed)	0.039	0.095	0.800	0.000
	*N*	674	736	697	1,010
Catastrophizing	Pearson correlation	0.121^**^	0.162^**^	0.089^*^	0.342^**^
	Sig. (two-tailed)	0.002	0.000	0.019	0.000
	*N*	674	736	697	1,010

**p < 0.01, *p < 0.05.

**Table 5:** Children, pre-teenagers and teenagers’ somatization and maladaptive coping strategies used by parents.

		somatization_children	somatizationpreteenagers	Somatization teenagers	Self-blame
somatization_children	Pearson correlation	1	−0.218^**^	−0.173^**^	0.118^**^
	Sig. (two-tailed)		0.000	0.000	0.000
	*N*	1,010	1,010	1,010	1,010
somatization_preteenagers	Pearson correlation	−0.218^**^	1	−0.217^**^	0.127^**^
	Sig. (two-tailed)	0.000		0.000	0.000
	*N*	1,010	1,010	1,010	1,010
somatization_teenagers	Pearson correlation	−0.173^**^	−0.217^**^	1	0.056
	Sig. (two-tailed)	0.000	0.000		0.076
	*N*	1,010	1,010	1,010	1,010
Rumination	Pearson correlation	0.081^**^	0.028	0.014	0.435^**^
	Sig. (two-tailed)	0.010	0.366	0.662	0.000
	*N*	1,010	1,010	1,010	1,010
Catastrophizing	Pearson correlation	0.097^**^	0.103^**^	0.075^*^	0.342^**^
	Sig. (two-tailed)	0.002	0.001	0.017	0.000
	*N*	1,010	1,010	1,010	1,010
blaming other	Pearson correlation	0.062	0.063^*^	0.059	0.179^**^
	Sig. (two-tailed)	0.050	0.047	0.061	0.000
	*N*	1,010	1,010	1,010	1,010

**p < 0.01, *p < 0.05.

**Table 6:** Children adaptability and maladaptive coping strategies used by parents.

		adaptability_children	adaptability_preteenagers	adaptability teenagers	catastrophizing
adaptability_ children	Pearson correlation	1	−0.413^**^	−0.320^**^	0.019
	Sig. (two-tailed)		0.000	0.000	0.544
	*N*	1,010	1,010	1,010	1,010
adaptability_ preteenagers	Pearson correlation	−0.413^**^	1	−0.474^**^	0.002
	Sig. (two-tailed)	0.000		0.000	0.954
	*N*	1,010	1,010	1,010	1,010
adaptability_ teenagers	Pearson correlation	−0.320^**^	−0.474^**^	1	−0.065^*^
	Sig. (two-tailed)	0.000	0.000		0.039
	*N*	1,010	1,010	1,010	1,010
Catastrophizing	Pearson correlation	0.019	0.002	−0.065^*^	1
	Sig. (two-tailed)	0.544	0.954	0.039	
	*N*	1,010	1,010	1,010	1,010

**p < 0.01, *p < 0.05.

Maladaptive coping strategies, namely self-blame, significantly raises the children’s depression level (*r* = 0.128, *p* = 0.000), that of the pre-teenagers (*r* = 0.120, *p* = 0.000), and that of the teenagers (*r* = 0.068, *p* = 0.030).

Rumination as maladaptive coping strategy was linked with children’s depression level, for ages between 2 and 6 years old (*r* = 0.121, *p* = 0.000), while blaming other links with the level of depression in small children (*r* = 0.075, *p* = 0.017). Catastrophizing links with children’s depression, (*r* = 0.063, *p* = 0.044), to that of pre-teenagers (*r* = 0.087, *p* = 0.006) and teenagers (*r* = 0.069, *p* = 0.028). The effect size index (*r*^2^) values range between 0.003 and 0.014.

Also, children’s anxiety links to all the maladaptive coping strategies: self-blame (*r* = 0.187, *p* = 0.000), blaming others (*r* = 0.079, *p* = 0.000), rumination (*r* = 0.164, *p* = 0.000), catastrophizing (*r* = 0.121, *p* = 0.002). Pre-teenagers’ anxiety links with self-blame (*r* = 0.160, *p* = 0.000) and catastrophizing (*r* = 0.162, *p* = 0.000), while teenagers’ anxiety is related to catastrophizing (*r* = 0.089, *p* = 0.019). *r*^2^ values are between 0.006 and 0.034.

Small children’s somatization links to the following maladaptive coping strategies: self-blame (*r* = 0.118, *p* = 0.000), rumination (*r* = 0.081, *p* = 0.010), and catastrophizing (*r* = 0.097, *p* = 0.002), pre-teen’s somatization links to self-blame (*r* = 0.127, *p* = 0.000), blaming others (*r* = 0.063, *p* = 0.047) and catastrophizing (*r* = 0.103, *p* = 001), while teenagers’ somatization links to catastrophizing (*r* = 0.075, *p* = 0.17). The coefficient of determination *r*^2^ values are between 0.003 and 0.016.

The more the level of catastrophizing increases, the more the teenagers’ adaptability level decreases (*r* = −0.065, *p* = 0.039, *r*^2^ = 0.004).

### 3.3 Family resilience

Family resilience, through the dimension of ‘communication and problem-solving in the family’ has a moderation role in the relationship between parental depression and the maladaptive coping strategy of rumination. One can notice that *p*-value for interaction is lower than 0.001 (Table 7), and *F* = 60.298 (Table 8). We chose the communication and problem-solving in the family dimension as a moderator because it is part of Walsh’s model (2015) of family resilience, which we followed in the study.

**Table 7:** The moderator effect of family resilience (Communication and problem-solving dimension) in the relationship between depression and rumination (interaction effect).

Model		Unstandardized coefficients		Standardized coefficients	*t*	Sig.
		B	Std. error	Beta		
	(Constant)	4.356	0.211		20.624	0.000
	Rumination	−0.230	0.009	−0.197	−25.311	0.000
	Communication and problem-solving in the family	−0.006	0.002	−0.025	−3.292	0.001
	Interaction	0.061	0.000	1.015	128.448	0.000

**Table 8:** The moderator effect of family resilience (Communication and problem-solving dimension) in the relationship between depression and rumination.

**Model**		**Sum of squares**	**df**	**Mean square**	***F***	**Sig.**
	Regression	2026.263	2	1013.132	60.298	0.000
	Residual	16919.594	1007	16.802		
	Total	18945.857	1009			

Family resilience through ‘maintaining a positive attitude’ has a moderation role in the relationship between catastrophizing and the teenagers’ level of adaptability. The p-value for interaction is *p* < 0.001 (Table 9), and *F* = 2179.173 (Table 10).

**Table 9:** The moderation role of family resilience (dimension Maintaining a positive attitude) in the relationship between maladaptive coping (catastrophizing) and the level of adaptability for teenagers (interaction effect).

Model		Unstandardized coefficients		Standardized coefficients	*t*	Sig.
		*B*	Std. error	Beta		
	(Constant)	2.472	0.517		4.784	0.000
	Catastrophizing	−0.373	0.021	−0.209	−17.819	0.000
	Maintaining a positive attitude	0.055	0.018	0.036	3.090	0.002
	Interaction	0.100	0.001	0.937	80.190	0.000

**Table 10:** The moderation role of family resilience (dimension Maintaining a positive attitude) in the relationship between maladaptive coping (catastrophizing) and the level of adaptability for teenagers.

**Model**		**Sum of Squares**	**df**	**Mean Square**	***F***	**Sig.**
	Regression	34119.214	3	11373.071	2179.173	0.000
	Residual	5250.300	1006	5.219		
	Total	39369.514	1009			

## 4 Discussion

An empirical foundation for evaluating successful relationships and family functioning has been established in recent decades by systems-oriented family process research (Lebow and Stroud, 2012). However, family scales and typologies are often static and a contextual, providing a snapshot of interaction patterns but rarely relating them to a family’s stressors, resources, and challenges over time and in their social environment. Families most frequently seek assistance at times of crisis, when discomfort and deviations from the norm are all too easily interpreted as indicators of a dysfunctional family.

The study’s findings demonstrate the function and importance of family resilience within the family system and how a moment of crisis might influence this system. The study is congruent with the results of other research in which we are presented with the fact that it is critical to track coping techniques and classify the best coping mechanisms (Marchetti et al., 2020; Adams et al., 2021; Calvano et al., 2021). A classification even of the maladaptive coping mechanisms is necessary to be able to do family screening and find techniques to optimize the quality of life in times of crisis.

This study indicates that parents with maladaptive coping strategies are more affected by the pandemic, therefore the high scores on the subscales which measure maladaptive coping indicate high scores of the levels of depression, anxiety, and stress. The tendency to associate higher levels of parental stress with higher levels of anxiety and depression is consistent with the research that suggests a connection between parental stress and symptoms of anxiety and depression, independent of the pandemic (Pripp et al., 2010; Crugnola et al., 2016; Vismara et al., 2016; Rollè et al., 2017; Brown et al., 2020; Russell et al., 2020; Spinelli et al., 2020).

Therefore, the maladaptive coping strategies most often used and associated with emotional symptoms in children and adolescents are self-blame, catastrophizing, rumination and blaming others. The results indicate that the use of these strategies by parents is related to the emotional difficulties of children and adolescents such as depression, anxiety, and somatization.

Regarding adaptive coping strategies, they are associated with the level of adaptability of adolescents, a fact also supported by Rodriguez et al. (2014) in their study on coping, the family environment, and the emotional health of adolescents, in which findings involve the role of coping in the relationship between family environment and adolescent mental health.

The result according to which family resilience is a moderator in the relationship between depression and rumination is very valuable and shows us that family support plays an essential role in the way psychological stress is perceived and manifested during a crisis. This result is also supported by previous studies that show that family environment is an important moderator in the relationship between depression treatment and adolescent’s emotional difficulties (Dardas, 2019).

Spending time with parents can effectively reduce the symptoms of adolescents with depression (Manczak et al., 2019). Vladislav et al. (2022) emphasize the role of functional coping strategies, such as support strategies, in which social relationships and emotional adjustment predominate, in increasing adolescents’ self-confidence in the context of the COVID-19 pandemic. In fact, the results support the relationship with parents and social support in times of crisis.

Furthermore, family resilience has been identified as an effective family-centered intervention to promote family communication to improve the suffering of parents and children, and these improvements are sustained even after the intervention is discontinued (Saltzman, 2016).

The two subscales of family resilience used by us have not been followed in other studies, and the role of positive attitude and communication and problem solving within the family during periods of crisis is still not fully understood.

We consider that these two dimensions of family resilience bring some implications in the way to face difficult situations both for the individual and for the family. They can be the basis of family therapy in times of crisis, starting from the promotion of these two dimensions, as well as at the end of the therapeutic approach, being the therapeutic objectives for organizing the family in times of crisis.

The results of our study are congruent with those of Perry et al. (2023) who consider the fact that from a family resilience perspective, the COVID-19 pandemic required responses from the family system as roles, expectations, and relationships changed for all family members. Specifically, some families may be more vulnerable than others due to pre-pandemic factors, such as parent and child mental and physical health needs, and a history of trauma.

Therefore, the research will have an impact on the scientific, social, economic, and cultural environment. At the scientific level, the impact consists in the dissemination of important results for developmental psychology, family psychology and psychotherapy, with implications for the emotional health of parents and children. At the social level, the impact consists in raising awareness at the level of communities and society regarding parents’ role in children’s emotional difficulties.

Regarding the economic environment, the impact consists of the fact that by disseminating the results of the study and raising awareness of the role of parents’ coping strategies in children’s emotional state, prevention, and early intervention programs for children and families can be carried out. This fact reduces the costs of more expensive treatments.

The impact on the cultural environment reflects promoting a culture centered on the family needs and experiences of children and their families and supporting a culturally and ethnically diverse approach to family care services.

### 4.1 Limitations and future research directions

There were several limitations to this investigation. First, because this study employed cross-sectional data to conduct a variety of intermediate effect analyses, we were unable to identify whether the factors were causally related. Consequently, to confirm the findings of this research, a comparable effect analysis should be performed using longitudinal data in the future.

Another limitation of this study was the absence of standardized, homogeneous patient groups for screening purposes. The emphasis on creating homogeneous groups of patients for screening suggests a recognition of the need for standardized approaches and methods in coping and family resilience screening. This aligns with the state-of-the-art, as it emphasizes the importance of consistency and comparability in research and clinical evaluations. Also, there is no data on the financial level of the families/ parents investigated, which may influence the level of resilience and coping strategies in a crisis (such as the COVID-19 pandemic). Further studies are needed to investigate family-level resilience and children’s emotional difficulties in a crisis, only in the context of higher and lower income families.

## 5 Conclusion

To the best of our knowledge, this is the first study that addresses the issue of children’s emotions during the pandemic and the coping methods used by their parents to deal with family difficulties that arise during a crisis. There is another study published by Pereira et al. (2023) that addresses child mental health during a crisis. A novelty factor is the inclusion of the family resilience variable in our research. We wanted to study the importance of family resilience in the adaptation of parents and children/adolescents in situations of adversity. The current research adds to the limited body of literature concerning parental coping strategies and their relationship to children’s emotional difficulties and adaptability.

The present study is oriented toward practical implications in the sphere of family therapy and the well-being of the family in times of crisis. The results indicate that maladaptive coping strategies of parents (catastrophizing, rumination, blaming) are associated with emotional difficulties in the case of children, pre-adolescents, and adolescents.

It is very important that parents are helped through psychological counseling programs to reduce these maladaptive coping strategies and to develop adaptive coping and stress management skills. Another practical implication is the development of work programs with families to improve family resilience and educate families to develop communication, receptivity and emotional availability of family members to each other, solving problems and increasing the positive attitude in the family.

## Data availability statement

The raw data supporting the conclusions of this article will be made available by the authors, without undue reservation.

## Ethics statement

The University of Bucharest’s Ethics Commission regulations were followed in conducting this study; registration number: 21/21.04.2020. The studies were conducted in accordance with the local legislation and institutional requirements. The participants provided their written informed consent to participate in this study.

## Author contributions

EV: Conceptualization, Data curation, Formal analysis, Investigation, Methodology, Supervision, Writing – original draft, Writing – review & editing. GM: Conceptualization, Data curation, Formal analysis, Investigation, Methodology, Resources, Writing – original draft, Writing – review & editing. C-IP: Conceptualization, Data curation, Formal analysis, Investigation, Methodology, Writing – original draft, Writing – review & editing. OP: Data curation, Methodology, Software, Writing – original draft.
